# Genetic variation in photosynthesis: many variants make light work

**DOI:** 10.1093/jxb/erac129

**Published:** 2022-05-23

**Authors:** Johannes Kromdijk, Alistair J McCormick

**Affiliations:** 1 Department of Plant Sciences, University of Cambridge, Downing Street, Cambridge CB2 3EA, UK; 2 Carl R Woese Institute for Genomic Biology, University of Illinois at Urbana-Champaign, 1206 W Gregory drive, Urbana, IL 61801, USA; 3 SynthSys & Institute of Molecular Plant Sciences, School of Biological Sciences, University of Edinburgh, Edinburgh EH9 3BF, UK

**Keywords:** C_3_ photosynthesis, C_4_ photosynthesis, CO_2_ assimilation, genetic variation, plant breeding, biotechnology

**Advances in breeding practices during the ‘Green Revolution’ in the 1960s have helped to maintain crop yields in the face of population growth. However, finding opportunities for further improvements is increasingly challenging, particularly due to the negative impacts of climate change (** Dusenge *et al.*, 2019 **). Solutions to this challenge must be found in new technologies, including new genetic engineering strategies, high-throughput phenotyping approaches, and greater exploitation of the available genetic variation within crop germplasms. Traditional breeding practices have yet to be exploited efficiently for the production of significant improvements in crop photosynthetic efficiencies. Achieving this in concert with new technologies is now a major goal.**

## Natural genetic variation in photosynthesis as a tool to improve understanding

Genetic variation between or within species provides a record of selection pressure over evolutionary time scales. Climatic conditions form an important component of such environmental selection pressures. Although photosynthetic traits can acclimate to prevailing climatic conditions during growth and development, innate ‘hard-coded’ differences based on genomic sequence diversity can greatly aid species adaptation to a particular environment. Thus, characterizing genetic diversity in photosynthetic traits can help to enhance our understanding of factors involved in adaptation, as well as providing a wealth of resource diversity that can be explored for industrial or agronomic applications.

Cyanobacteria are an evolutionary ancient group of highly diverse and environmentally adaptable microorganisms, also of notable fame for their engulfed group member that formed the evolutionary ancestor of the eukaryotic chloroplasts (Rockwell *et al.*, 2014). Despite high diversity within the cyanobacterial phylum, most of the work to understand their ecology, physiology, metabolism, and regulatory pathways, as well as their suitability as biotechnology chassis, has traditionally focused on a small number of model species. However, as Selão (2022) describes in this issue, substantial efforts to isolate and characterize new strains are now starting to expand the genetic diversity available for basic research and development of biotechnological applications.

An intriguing example of adaptation to suppress photorespiration is the convergent evolution of the C_4_ photosynthetic pathway. The C_4_ pathway involves a carbon-concentrating C_4_ acid shuttle between two specialized photosynthetic cell types, bundle sheath and mesophyll, which are typically organized in two concentric rings around the vascular bundles. The decarboxylation of C_4_ acids raises the CO_2_ concentration around the site of Rubisco accumulation in the bundle sheath cells, which suppresses Rubisco oxygenase activity and photorespiration. Sage *et al.* (2011) estimated that the C_4_ pathway has evolved independently at least 60 times. The global distribution of C_4_ photosynthesis is often associated with habitats that are conducive to high rates of photorespiration (Ehleringer *et al.*, 1997). However, using geographical distribution data of C_3_ and C_4_ tree species in the *Euphorbiaceae*, Young *et al.* (2022) now show that the unique presence of C_4_ photosynthesis in trees from this family does not conform to this general pattern, but may instead have allowed expansion of the ecological niche into higher elevation habitats with cooler temperatures.

The productivity benefits of the C_4_ pathway have inspired major bioengineering efforts to install the pathway into C_3_ crop species such as rice, progress of which was recently reported by Ermakova *et al.* (2020). Understanding of the genetic mechanisms leading to the formation of C_4_ anatomy and biochemistry is paramount to making this feasible. In this issue, Simpson *et al.* (2022) review attempts to make use of hybridization between C_3_ and C_4_ species, or within species between accessions with contrasting C_4_ traits, to study the photosynthetic characteristics of the progeny. They also make a strong case for the use of artificial selection and genome-wide association to speed up discovery of genetic determinants underpinning the C_4_ syndrome.

## Leveraging natural genetic variation in photosynthesis to improve crop performance

Genetic variation is the key ingredient for improving crop performance via selective breeding. In some crops, comparisons between historical cultivars sorted by release date have provided evidence of selection on photosynthesis traits as an inadvertent side effect of plant breeding efforts. For example, Koester *et al.* (2016) reported that more recently released cultivars of soybean tended to show higher daily carbon gain than older cultivars, primarily via enhanced stomatal conductance in periods of high soil water content. In this issue, Li *et al.* (2022) suggest that breeding may also have affected photosynthetic characteristics of *Triticum aestivum* (wheat), based on a comparison of 26 winter wheat cultivars spanning 60 years of wheat breeding in China.

Whereas genetic variation in photosynthetic traits is significant in many major crop species (reviewed by Sharwood *et al.*, 2022 and Sakoda *et al.*, 2022 in this issue), incorporation of photosynthesis in selective breeding programmes is still rare. Theeuwen *et al.* (2022) discuss how quantitative genetics can be used to discover useful trait variation and design strategies to improve crop photosynthesis, based on readily available crop plant germplasm. Crop breeding strategies require a defined target population of environments (TPE), namely a variable group of future production environments (Crespo-Herrera *et al.*, 2021), under which the breeding programme attempts to enhance crop performance. The importance of clearly defined TPE for research to enhance crop photosynthesis is illustrated by Sales *et al.* (2022), who present a complete lack of correlation between glasshouse and field evaluation of photosynthetic traits across 80 wheat lines. Their work emphasizes the importance of genotype by environment interactions, especially for complex, highly multigenic traits. In the context of global climate change, the increased occurrence of extreme weather events (IPCC, 2021) means that the stochastic, unpredictable component of TPE is becoming more prominent and selection for enhanced resilience against stress is gaining importance. It should therefore be no surprise that genetic variation in photosynthetic traits in response to abiotic stress is strongly represented in the current issue. Faralli *et al.* (2022) present variation between stomatal dynamics across a range of *Vitis vinifera* (grapevine) genotypes, which contributed significantly to differentiation in heat tolerance and water use efficiency. Ortiz and Salas-Fernandez (2022) analyse the genetic control of photosynthesis in response to drought stress in *Sorghum bicolor*, identifying several genomic regions associated with variation in gas exchange and chlorophyll fluorescence traits, which might be used to further enhance already substantial drought tolerance of this C_4_ food, feed, and bioenergy crop. Posch *et al.* (2022) show that thermal tolerance of PSII in wheat is subject to rapid acclimation in response to short-term supraoptimal temperature conditions, but also varies significantly between a range of genotypes with contrasting high temperature tolerance. Finally, on the suboptimal end of growth season temperature, Burnett and Kromdijk (2022) argue the case for enhancing chilling tolerance of photosynthesis in maize via selective breeding, which may help to better adapt the global number one grain crop to its increasingly temperate surroundings as its cultivation area spreads further north.

## Tools to assess and utilize natural variation in photosynthesis

Although the germplasm of most crops already appears to show significant variation for photosynthetic traits, genetic diversity within a crop’s germplasm is expected to be significantly less than that of its wild progenitors. The negative effect of domestication on genetic diversity is well known, although the often-quoted ‘domestication bottleneck’ typically blamed for this phenomenon has recently become the subject of renewed scrutiny (Smith *et al.*, 2019). Regardless of the mechanism involved, it is clear that expanding allelic diversity can be a fruitful route to enhance breeding programmes. Sharwood *et al.* (2022) review the great potential to find novel allelic variation in photosynthetic traits in large germplasm collections for food and fibre crops and their wild relatives, and how this variation could be leveraged to accelerate genetic progress in crop breeding programmes. Research on *Oryza glaberrima* (African rice) by Cowling *et al.* (2022) provides a case in point. Whereas African rice is not suitable for commercial rice production due to lodging, shattering, and low yield, it makes an attractive target for gene mining for introgression into *O. sativa indica* and *japonica*. Having been domesticated independently from *O. sativa*, African rice germplasm harbours many traits that help to sustain plant growth under challenging conditions. Indeed, Cowling and colleagues show that under common growth conditions, extensive variation in many steady-state and dynamic photosynthetic traits across a reference panel of 155 accessions clustered in line with the prevailing climate conditions of their collection sites, in particular water availability.

As emphasized by Sharwood *et al.* (2022), a major challenge still resides in phenotyping of large collections of diverse germplasm, which can quickly become prohibitively expensive. However, methods to enable high-throughput phenotyping for photosynthesis under field conditions have made significant strides driven by advances in sensor technology and statistical techniques, and are starting to make an impact, providing proxies for photosynthetic traits rapidly enough to cover field experiments with several hundred accessions. Fu *et al.* (2022) review the utility of high-throughput fluorescence and hyperspectral techniques to estimate photosynthetic traits from leaf to canopy and infer six key lessons learned with regards to precision, scalability, standardization, and overall utility. Whereas these techniques are easing the phenotyping bottleneck to some degree, it is difficult to see how phenotyping capacity could ever become sufficient to screen large germplasm collections consisting of tens of thousands of accessions. This is where Sharwood *et al*. recommend a ‘genome to phenome’ approach, using accession-specific sequence information to find haplotypes for key candidate genes in photosynthesis. Models to integrate physiological and accession-specific genetic information are a key ingredient of this approach. Linking of model parameters directly to underlying genetic variation can be very powerful to assess the potential impact of allelic variants across a range of genetic loci on crop performance across the TPE. A striking example of such an approach is provided by seminal work from Messina *et al.* (2020, Preprint) on model-aided breeding of drought-tolerant maize, which approximately doubled the rate of crop improvement under drought. While the genetic mechanisms that control many parameters in the photosynthesis module of crop models are not yet sufficiently understood to mimic this approach, Yin *et al.* (2022) take a first step in this direction by reviewing how photosynthetic model parameters have been observed to vary within germplasm of C_3_ crop species. Using their GECROS model, they go on to show the relative importance of genetic variation in model parameters describing photosynthetic electron transport, relative to parameters controlling the light-saturated rate of CO_2_ assimilation, such as maximum Rubisco activity. Their simulations also predict that breeding programmes incorporating selection for traits to improve electron transport, nitrogen uptake, and sink capacity would provide the highest impact on yield.

Overall, the new strategies and technologies highlighted in the Special Issue are poised to provide a step change in our capacity to breed new crop varieties with improved photosynthetic traits and greater resilience. Such new tools should provide timely support to an agricultural industry under huge pressure to meet future food security demands.
